# Disorder-specific characteristics of borderline personality disorder with co-occurring depression and its comparison with major depression: An fMRI study with emotional interference task

**DOI:** 10.1016/j.nicl.2016.08.015

**Published:** 2016-08-21

**Authors:** Natalia Chechko, Thilo Kellermann, Marc Augustin, Michael Zvyagintsev, Frank Schneider, Ute Habel

**Affiliations:** Department of Psychiatry, Psychotherapy and Psychosomatics, RWTH Aachen University, Pauwelsstr. 30, D-52074 Aachen, Germany; JARA — Translational Brain Medicine, Forschungszentrum Jülich GmbH, D-52425 Jülich, Germany

**Keywords:** Borderline personality disorder, major depression disorder, Emotional interference task, Functional magnetic resonance imaging, Lateral prefrontal cortex, Extrastriate visual cortex

## Abstract

Borderline personality disorder (BPD) and major depressive disorder (MDD) are both associated with abnormalities in the regulation of emotion, with BPD being highly comorbid with MDD. Disorder-specific dysfunctions in BPD, however, have hardly been addressed, hence the lack of knowledge pertaining to the specificity of emotion processing deficits and their commonality with MDD.

24 healthy comparison subjects, 21 patients with MDD, and 13 patients with comorbid BPD and MDD (BPD + MDD group) were studied using functional MRI. The subjects were required to perform an emotional interference task that entailed categorizing facial affect while ignoring words that labeled the emotional contents of the external stimuli.

Collapsing across emotional face types, we observed that participants with BPD + MDD uniquely displayed a greater involvement of the visual areas and the cerebellum. During emotional conflict processing, on the other hand, the lateral prefrontal cortex (LPFC) appeared to be affected in both patient groups. In comparison to the HC, the MDD group showed differences also in the posterior medial frontal cortex (pMFC) and the inferior parietal lobule (IPL).

Thus, our data indicate dysfunctionality in the neural circuitry responsible for emotional conflict control in both disorders. The enhanced visual cortex activation in BPD + MDD suggests the visual system's hyperresponsiveness to faces at an early perceptual level. Not being associated with co-occurring depression, this effect in BPD + MDD appears to represent specific personality traits such as disturbed reactivity toward emotionally expressive facial stimuli.

## Highlights

• 24 healthy controls, 21 patients with MDD, and 13 patients with BPD + MDD were studied using fMRI
• During emotional conflict processing, the lateral prefrontal cortex (LPFC) was affected in both patient groups
• BPD + MDD group uniquely displayed a greater involvement of the visual areas and the cerebellum

## 1 Introduction

Borderline personality disorder (BPD) is a severe mental disorder with a 5.9% prevalence rate in the general population (Grant et al., 2008). The condition is characterized by pronounced deficits in emotion regulation as well as cognitive disorders including dissociation, impulsivity, and interpersonal and social disturbances (Herpertz et al., 2014, Krause-Utz et al., 2014, Skodol et al., 2002). BPD is also highly comorbid with depression, anxiety, substance-use and other personality disorders (PDs). According to most clinical studies, of all conditions co-occurring with BPD, concomitant depressive disorder (MDD) has the highest (up to 75%) lifetime prevalence rate (Grant et al., 2008).

Disorders frequently co-occur when they share some biological features (Koenigsberg et al., 1999). Neuroimaging studies have shown that both BPD and MDD are associated with dysfunction of the prefrontal cortex coupled with amygdala hyperactivity (Drevets et al., 2008, Herpertz et al., 2014, Krause-Utz et al., 2014, Siegle et al., 2007). However, even while co-occurring, the conditions often have independent clinical courses. For instance, depression in the context of borderline personality disorder has been seen to predict a worse response to antidepressants in general (Beatson and Rao, 2012), whereas prior improvements in BPD, on the other hand, can predict improvements in MDD, although not vice versa (Gunderson et al., 2004).

Direct comparisons of neuroimaging data obtained from BPD and MDD can help delineate the disorder-specific as well as shared characteristics. Given the high prevalence of depression among BPD patients, controlling for co-occurring affective conditions can additionally help identify disorder-specific features. The problems common to both disorders include affect regulation of emotions and thoughts, and, therefore, understanding the relationship between brain networks involved in the execution of control and determination of the affective value of stimuli is of utmost importance. Emotional distractors have been proved to be particularly effective in capturing our attention and processing resources. As the emotional Stroop task requires inhibition of interference by emotional distractors while a cognitive task is being performed, it is particularly conducive to a comprehensive comparison between MDD and BPD.

Several neuroimaging studies have investigated the neural correlates of response inhibition in BPD by means of an emotional Stroop paradigm with subjects being asked to identify the ink color of words that are either emotionally neutral or emotionally salient (Malhi et al., 2013, Wingenfeld et al., 2009). The slowing of reaction times for color naming of emotional words relative to neutral words served as a measure of emotional interference effect (Compton et al., 2003, Williams et al., 1996). This version of the emotional Stroop task, however, does not directly assess the interference of emotion processing with cognitive processing, as the meaning of the emotional word is semantically unrelated to the task-relevant information (Etkin et al., 2006) and thus lacking a stable interference effect. The lack of a reliable behavioral interference in this version of the emotional Stroop task, therefore, limits the conclusions drawn in relation to abnormalities in the prefrontal and limbic systems in BPD (Malhi et al., 2013, Wingenfeld et al., 2009).

In light of these observations, we sought to expand upon our previous findings, obtained through an emotional Stroop-like task (emotional interference task) based on the semantic conflict between emotional distractor (emotional words) and targets (faces), by focusing, in the current study, primarily on BPD with co-occurring depression (BPD + MDD group) and its comparison with MDD. The chosen version of the emotional interference task involves recognition of facial affect (fearful, sad or happy expressions) while ignoring the overlaid words “fear”, “sad” or “happy” (Chechko et al., 2012, Chechko et al., 2013). In our previous studies, the triggering of a stable behavioral conflict between incongruent and congruent trials was seen to lead to a robust activation of neural network including the posterodorsal medial frontal cortex (pMFC), the bilateral anterior insula, the bilateral lateral prefrontal cortex (LPFC) and the extrastriate visual cortex (Chechko et al., 2012, Chechko et al., 2013, Chechko et al., 2014). In addition, in MDD, a comparison with heathy controls revealed hypoactivation in the ventrolateral prefrontal, parietal and extrastriate cortices (Chechko et al., 2013).

Applying this version of emotional Stroop task to healthy controls, the BPD + MDD group and the group of MDD patients reported before (Chechko et al., 2013), we expected to see group differences in regions responsible for emotional conflict regulation. Furthermore, by focusing primarily on BPD with co-occurring depression, we expected to be able to determine (through comparison with MDD) the abnormalities linked to the psychopathology of BPD as well as those associated with the co-occurring affective disorder.

## 2 Methods and materials

### 2.1 Participants

The study involved 34 patients, all of whom were recruited during their stay at the Department of Psychiatry, Psychotherapy and Psychosomatics, University Hospital Aachen, and 24 healthy controls recruited through advertisements.

The diagnostic assessments were made by a single rater and an experienced psychiatrist (NC) using both an informal clinical interview and the German version of the Structured Clinical Interview for DSM-IV (Axis I and Axis II disorders) (Fydrich et al., 1997, Wittchen, 1997). The exclusion criteria were bipolar and psychotic disorders, current substance use, drug or alcohol dependency, posttraumatic stress disorder, history of a neurological disorder, head trauma or loss of consciousness, and claustrophobia. Patients with a past alcohol or drug abuse history were required to be abstinent for at least 4 weeks.

The depression-only group (*N* = 21) consisted of patients in whom MDD was the primary diagnosis without any comorbidity, while the BPD group (*N* = 13) was made up of patients with comorbid MDD (BPD + MDD group). All patients with BPD + MDD met the criteria for current moderate to severe depressive symptoms.

As regards other axis I disorders in the BPD + MDD group, there were two patients with a past history of alcohol abuse, one patient with a history of drug (cannabis and amphetamine) and alcohol abuse, two patients with cannabis abuse, and four with a current eating disorder (one with bulimia nervosa, one with an eating disorder not otherwise specified, one with binge eating disorder, and one with anorexia nervosa).

All BPD + MDD patients were treated either with antidepressant monotherapy or combination therapy involving antidepressants and mood-stabilizing drugs (atypical antipsychotics or anticonvulsants), with 77% of MDD patients receiving antidepressants as monotherapy or in combination with other antidepressants.

Seven patients (50%) in the BPD + MDD and 8 patients (38%) in the MDD groups reported a history of interpersonal trauma exposure including emotional maltreatment (e.g., neglect, emotional abuse), physical abuse, and/or sexual abuse. None of the patients met the diagnostic criteria for current PTSD.

Symptom severity in the MDD group was assessed with the Hamilton Rating Scale for Depression (21-item version) (Hamilton and White, 1960). On the day of measurement, the severity of symptoms in all groups was also assessed based on the Becks Depression Inventory (BDI, (Beck et al., 1961), with BPD + MD patients being additionally subjected to the Clinical Personality Disorders Inventory (Andresen, 2006). All BPD + MDD patients showed very high (T-value 70; 4 participants) to extremely high (T-value 75; 9 participants) values on the Borderline Personality Disorder scale.

24 healthy participants were screened for neurological, psychiatric or other medical illnesses with impact on brain functioning (SCID-I, SCID-II, German version (Fydrich et al., 1997, Wittchen, 1997). None of the healthy comparison subjects had any current or past axis I or II disorders and took any psychiatric medication.

There were no differences between the two patient groups in the severity of depressive symptoms assessed by BDI (p = 0.23; 31.0 ± 8.2 vs. 33.6 ± 8.1 in BPD + MDD ‘s vs. MDD patients). The MDD patients were significantly older than healthy controls (p < 0.001; 36.5 ± 10.8 vs. 26.6 ± 3.2) and the patients with BPD + MDD (p < 0.001; 36.5 ± 10.8 vs. 24.6 ± 5.0). In addition, the BPD + MDD patients were significantly younger at the time of onset of the first depressive episode (p < 0.001; 18.1 ± 3.3 vs. 29.7 ± 7.9 in BPD + MDD's vs. MDD patients). The demographic and clinical characteristics are outlined in detail in Table 1. Data pertaining to the emotional conflict of healthy subjects and 18 of the depressed-only patients have been reported elsewhere (Chechko et al., 2012, Chechko et al., 2013).

### 2.2 FMRI paradigm

**Fig. 1:**
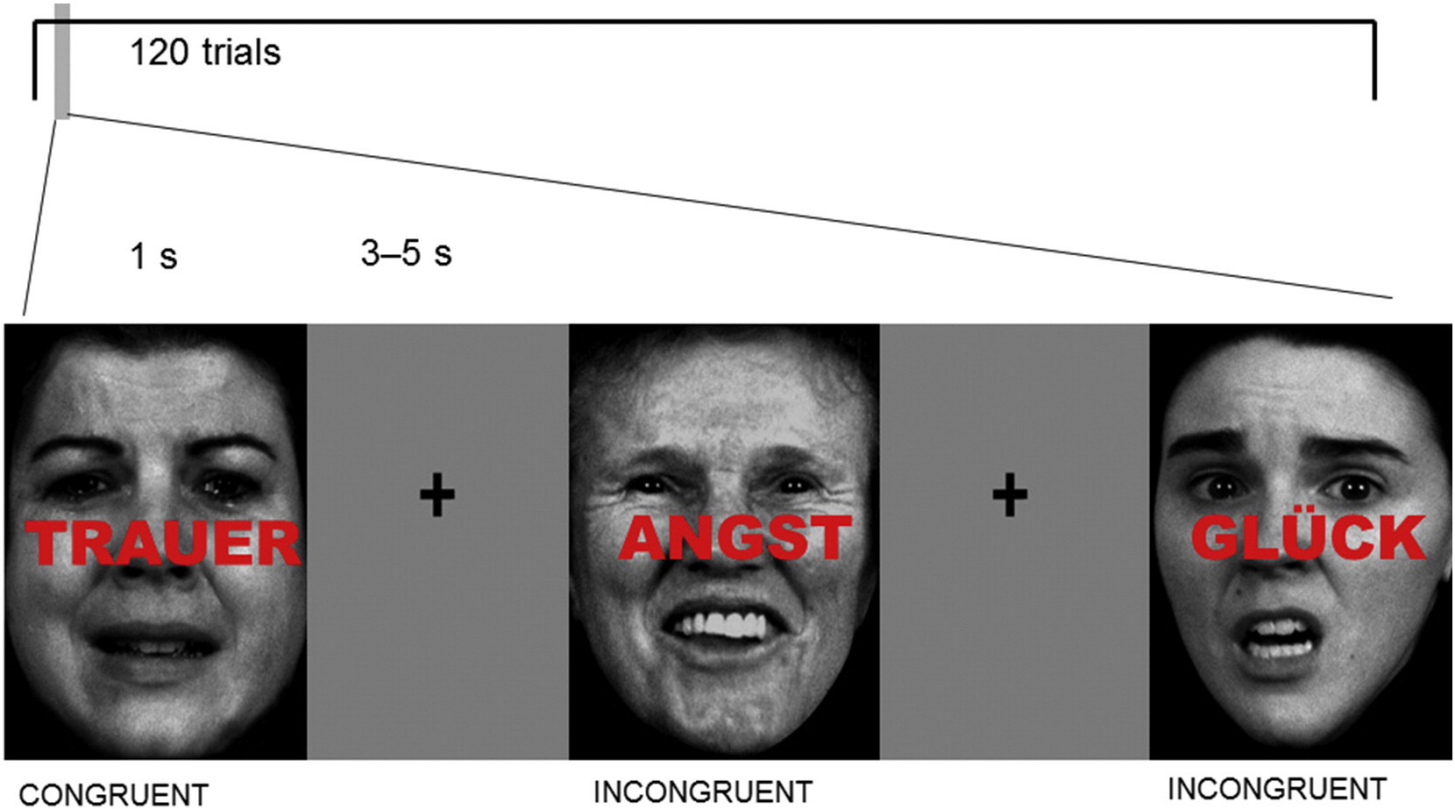
Emotional Stroop paradigm Basic stimulus material consisting of congruent and incongruent face expression/word pairs from the FEBA face collection.

The emotional conflict task was performed as previously described (Chechko et al., 2012, Chechko et al., 2013). It comprised 120 presentations of photographs of happy, sad or fearful facial expressions, each overlaid with one of the following words (distractors) ‘TRAUER’, ‘ANGST’, and ‘GLÜCK’ (German for “sadness”, “fear” and “happiness”) (Fig. 1). The trials were classified as congruent (C) or incongruent (I), with the number of congruent and incongruent trials and the number of word-face combinations being counterbalanced. Trials were displayed for 1000 ms with randomized interstimulus intervals (4.00 ± 0.38 s, range 3–5 s) using Presentation software (Neurobehavioral Systems, San Francisco, USA). Between face presentations, a fixation cross was shown. Participants were instructed to identify the target and answer as quickly and precisely as possible, and they indicated facial affect by pressing one of the three answer buttons with the right index, middle or ring fingers for sad, fearful or happy faces respectively.

Images of the faces were derived from the set used in Facial Emotions for Brain Activation (FEBA) test (Gur et al., 2002) and placed in standardized positions of the eyes and the mouth and normalized brightness. Participants looked at the pictures via video goggles (VisuaStim XGA, Resonance Technology Inc., Los Angeles, USA) and gave responses via the LUMItouch response system (http://ucdirc.ucdavis.edu/techsupport/Lumitouch_brochure.pdf).

### 2.3 MRI data acquisition

Functional imaging was performed on a 3 T Trio MR scanner (Siemens Medical Systems, Erlangen, Germany) using echo-planar imaging sensitive to BOLD contrast (voxel size: 3.0 × 3.0 × 3.0 mm3, 64 × 64 matrix, FoV: 192 mm2, 34 slices, gap 0. 75 mm, TR 2 s, TE 28 ms, α = 77°). A 4 min magnetization-prepared rapid acquisition gradient echo image (MP-RAGE) T1-weighted sequence was used to acquire structural images (TR = 1900 ms, TE = 2.52 ms, TI = 900 ms, matrix = 256 × 256, 176 slices, FoV: 250 × 250 mm2, alpha = 9°, voxel size = 1 × 1 × 1 mm3).

### 2.4 Analysis of behavioral data

Reaction times (RT) were collected during the fMRI experiment. Error trials (wrong answers and omissions) were excluded from RT analysis, with all types of errors being considered for accuracy calculations. For a 3-way group × emotion × congruency analysis of variance (ANOVA) for RT and accuracy, items were assigned to each level of the factors congruency (two levels: congruent or incongruent), group (two levels: healthy subjects or patients) and emotions (three levels: sadness, fear, happiness).

### 2.5 FMRI data analysis

Images were processed using Statistical Parametric Mapping (SPM) software (version SPM5, http://www.fil.ion.ucl.ac.uk/spm). The first five images of each time series were excluded due to T1 stabilization effects. All remaining images were slice-time corrected and realigned to the first image. Images were normalized to a standard EPI template (interpolation to 2 × 2 × 2 mm3 resolution) and smoothed with an isotropic Gaussian kernel (8 mm full width at half maximum).

For each subject and task, a first-level model was estimated including six regressors of interest: 2 levels of congruency (congruent vs. incongruent) by the 3 levels of facial expression (sadness, fear and happiness). Delta functions with the time-points of trial presentation of each type were convolved with the canonical hemodynamic response function (HRF) to build regressors for the time-series model. The first-level model included an additional (HRF-convolved) regressor of no interest for error trials (wrong answers and omissions) and an intercept for the mean across each session. A high-pass filter with a cut-off period of 128 s was applied and serial auto-correlations were accounted for by including a first-order auto-regressive covariance structure (AR(1)).

Contrast estimates of all regressors of interest from each subject were entered in a four-way ANOVA with independent observations of the factor group and repeated measures on the factors congruency and facial expression. The resulted columns in the design matrix.

represented HC, MDD and BPD + MDD groups. Thus, on the second level, the sessions were modeled with 18 regressors of interest: 3 groups (BPD + MDD, MDD and HC) by 2 trial types (congruent vs. incongruent) by 3 categories of facial expression of emotion.

At the group level, functional images were analyzed with t-contrasts comparing all incongruent trials with all congruent trials for each group separately. The statistical threshold, unless noted otherwise, was set at p < 0.05 FWE (family-wise error)-corrected on the voxel level, applying a cluster-extent threshold of 10 voxels. In the next step, the groups were compared with respect to brain networks involved in the emotional conflict. For the between-groups comparison, since we did not find any significant effect at p < 0.05 FWE-corrected on the voxel level, results were p < 0.05 FWE-corrected on the cluster level with a cluster-forming threshold of p < 0.001. The same statistical threshold was used for the correlational analyses, which were performed to determine the relationship between activation strength in response to incongruent (as compared to congruent) trials in single-subject contrasts on the one hand, and individual symptom severity (Hamilon score or BDI score) or individual behavioral interference effect on the other.

For a 2-way group × congruency analysis of variance (ANOVA), items were assigned to each level of the factors congruency (two levels: congruent or incongruent) and group (two levels: healthy subjects or patients). Again, results were p < 0.05 FWE-corrected on the cluster level with a cluster-forming threshold of p < 0.001.

## 3 Results

### 3.1 Behavioral data

A 3-way *group* × *emotion* × *congruency* RT analysis of variance (ANOVA) revealed significant effects of the factor *congruency* (*F*_1,24_ = 124.66, p < 0.001, ɳ^2^ = 0.9) and the factor *emotion* (F_2,24_ = 36.53, p < 0.001, ɳ^2^ = 0.75), with the factor *group* (F_2,24_ = 3.11, p = 0.063, ɳ^2^ = 0.21) missing the significance threshold only by a small margin. Thus, the emotional distractor expectedly induced a slowdown in responses in all groups during incongruent trials: t_23_ = − 9.97, p < 0.001: 1042 ± 206 ms vs. 905 ± 168 ms (mean ± SD) in the control group, t_12_ = − 2.80, p = 0.016: 1174 ± 262 ms vs. 1109 ± 261 ms (mean ± SD) in the group of BPD + MDD patients and t_20_ = − 4.56, p < 0.001: 1266 ± 380 ms vs. 1146 ± 314 ms (mean ± SD) in the depression-only group. The size of interference effect defined as RT difference between incongruent and congruent trials was 138 ms, 120 ms and 65 ms for HC, MDD and BPD + MDD respectively. However, the *group* × *congruency* interaction effect (F_2,24_ = 2.31, p = 1.21, ɳ^2^ = 1.61) was not significant.

Compared to the control group, both patient groups performed more slowly: t_43_ = − 2.88, p = 0.006; 974 ± 185 msec vs. 1206 ± 343 msec (mean ± SD) for comparison and MDD groups respectively) and (t_35_ = − 2.29, p = 0.028; 974 ± 185 msec vs. 1141 ± 259 msec (mean ± SD) for comparison and BPD + MDD groups respectively.

There were no RT differences between the patient groups.

A 3-way group × emotion × congruency accuracy analysis of variance (ANOVA) revealed significant effects of the factors *congruency* (*F*_1,24_ = 11.52, p = 0.005, ɳ^2^ = 0.49) and *emotion* (F_2,24_ = 23.31, p < 0.001, ɳ^2^ = 0.66). Consequently, the interference effect (2%, 1% and 3% for HC, BPD + MDD and MDD respectively) was also seen in the accuracy analysis (t_23_ = − 2.60, p = 0.016: 97% vs. 95% in the HC group, t_12_ = − 0.81, p = 0.436: 94% vs. 93% in the group of BPD + MDD patients; t_20_ = − 3.15, p = 0.005: 96% vs. 93% in the MDD-only group), although it did not reach significance in the BPD + MDD group. Again, the *group* × *congruency* interaction effect (F_2,24_ = 1.95, p = 1.64, ɳ^2^ = 1.40) was not significant.

The overall performance of the BPD + MDD group was less accurate during emotional recognition as compared to the HC group (t_35_ = − 2.16, p = 0.038; 93.89% vs. 95.9% for BPD + MDD and HC groups respectively). There were no significant differences in accuracy either between the two patient groups or between the MDD and HC groups (94.3% vs. 95.9% for MDD and HC groups respectively).

In all groups, targets with happy facial expressions were processed faster (F_2,24_ = 36.53, p < 0.001; 1137 vs. 1168 vs. 952 angry, sad and happy faces respectively) and more accurately (F_2,24_ = 23.31, p < 0.001; 94% vs. 92% vs. 98% for angry, sad and happy faces respectively). And these effects were not influenced by the factor *group* (F < 1).

### 3.2 FMRI results

#### 3.2.1 Emotional interference effect (incongruent > congruent trials contrast) in HC

**Fig. 2:**
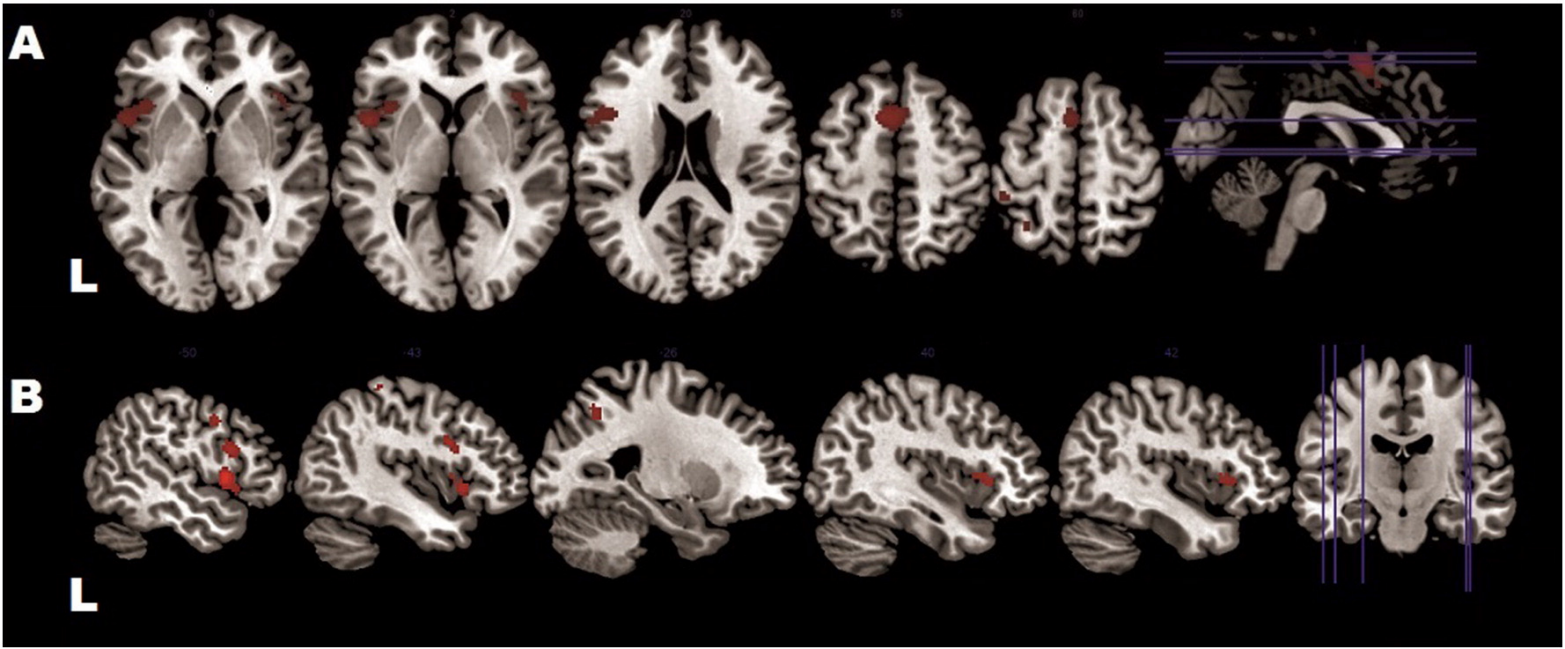
Areas recruited by emotional Stroop in controls A. Axial view: pMFC and bilateral VLPFC/anterior insula region B. Sagittal view: left LPFC and in the left posterior cortex Activity is shown at p < 0.05 family-wise error (FWE)-corrected on the voxel level with a cluster extent of > 10 voxels.

In the healthy controls, emotional conflict led reliably to activation in the bilateral inferior frontal gyrus (IFG) (right: *P. opercularis*; BA 44/45; peak MNI: 38/24/2; *T* = 5.38; 91 voxels, left: *P. opercularis* and orbitalis, BA 44/45; peak MNI: − 50/10/4; *T* = 6.76; 296 voxels), extending into the bilateral anterior insula (here referred to as VLPFC). Another large cluster of activation (329 voxels), referred to as the posterior medial frontal cortex (pMFC), encompassed the bilateral posterior medial frontal gyrus (peak MNI: − 6/10/56; *T* = 6.61; voxels = 329), the bilateral supplementary motor area (SMA, BA 6) and the pre-SMA, extending into the middle cingulate cortex (MCC). Activation was seen also in the parietal cortex (left: inferior parietal lobule; peak MNI:− 28/− 60/42; *T* = 5.25; voxels: 106; and left: superior parietal lobule; peak MNI:-30/− 54/64; *T* = 5.26; 28 voxels) (Fig. 2).

#### 3.2.2 Common and disorder-specific effects of emotional interference effect (incongruent > congruent trials contrast)

**Fig. 3:**
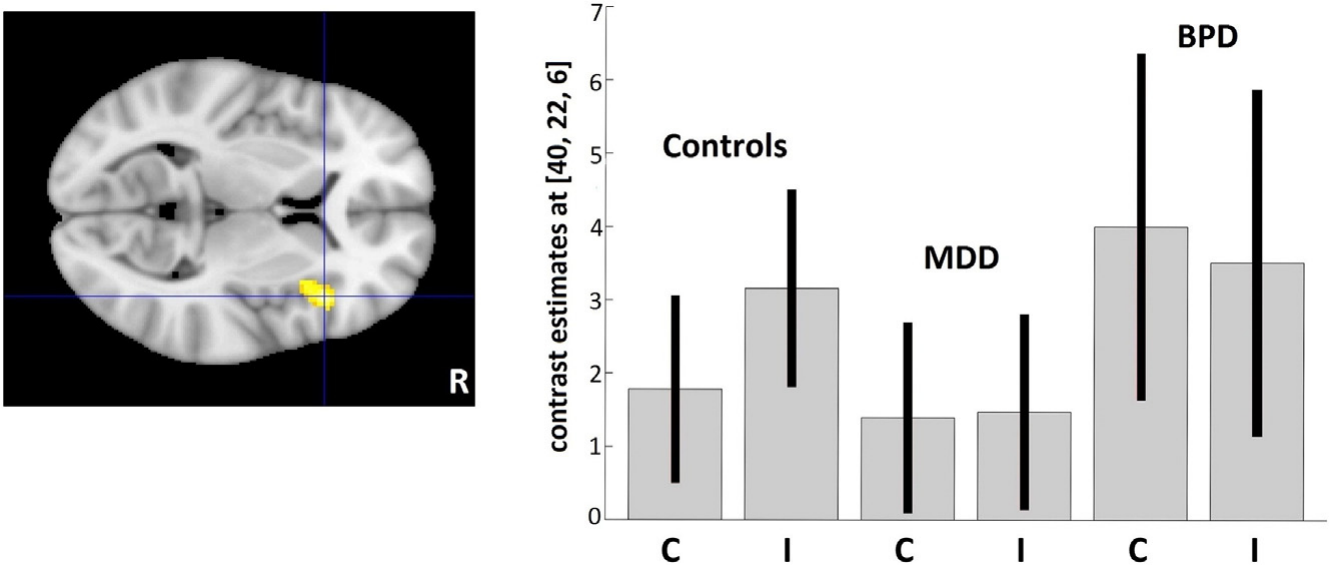
Significant effects of the *group* × *congruency* interaction in the right VLPFC/insula region Results are shown at p < 0.05 family-wise error (FWE)-corrected on the cluster level with cluster-forming threshold of p < 0.001.

The *group* × *congruency* interaction indicated differences in response to emotional conflict between the groups in the right VLPFC/insula region (*P. triangularis*; BA45 peak MNI: 40/22/6; F = 11.39, 161 voxels with p = 0.031 after FWE correction on the cluster level). The parameter estimates showed a stronger involvement of the VLPFC in response to incongruent (as compared to congruent) trials in HC, whereas this effect was missing in both patient groups (Fig. 3).

The between-groups comparison of the network related to interference effect (incongruent > congruent trials) showed differences between BPD + MDD and HC groups in the right VLPFC/insula region (peak MNI: 42/24/6; p = 0.008; *T* = 4.46; voxels: 284) (Fig. 3).

**Fig. 4:**
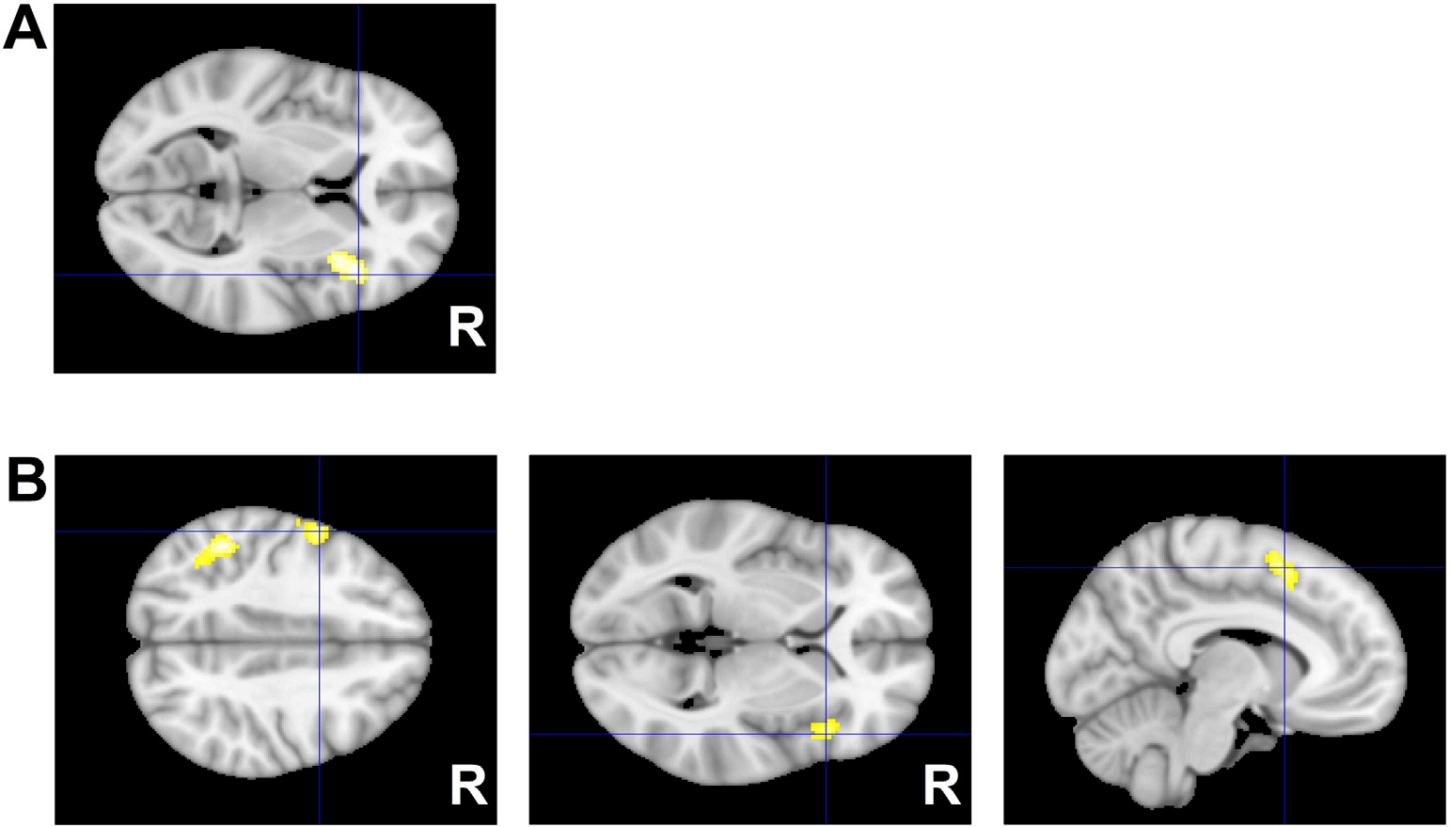
Areas recruited more weakly in BPD + MDD and MDD groups compared to controls in response to emotional Stroop A. Axial view: differences between patients and controls in the bilateral VLPFC/insula and left rolandic opperculum B. Sagittal view: differences between patients and controls in the pMFC Results are shown at p < 0.05 family-wise error (FWE)-corrected on the cluster level with cluster-forming threshold of p < 0.001.

The areas that were recruited significantly more weakly in MDD patients compared to the HC were the left operculum (peak MNI:-54/4/40; p = 0.003; *T* = 4.22; voxels: 349), the pMFC (peak MNI:-8/12/54; p = 0.004; *T* = 4.37; voxels: 322), the right VLPFC/insula region (peak MNI:46/20/2; p = 0.027; *T* = 4.07; voxels: 217), and the left inferior parietal lobule (peak MNI:− 46/− 44/38; p = 0.028; *T* = 4.85; voxels: 216). There were no significant differences between the patient groups, nor were there any relevant regions in which patients showed stronger activation compared to the HC (Fig. 4).

#### 3.2.3 Effect of emotional face processing

**Fig. 5:**
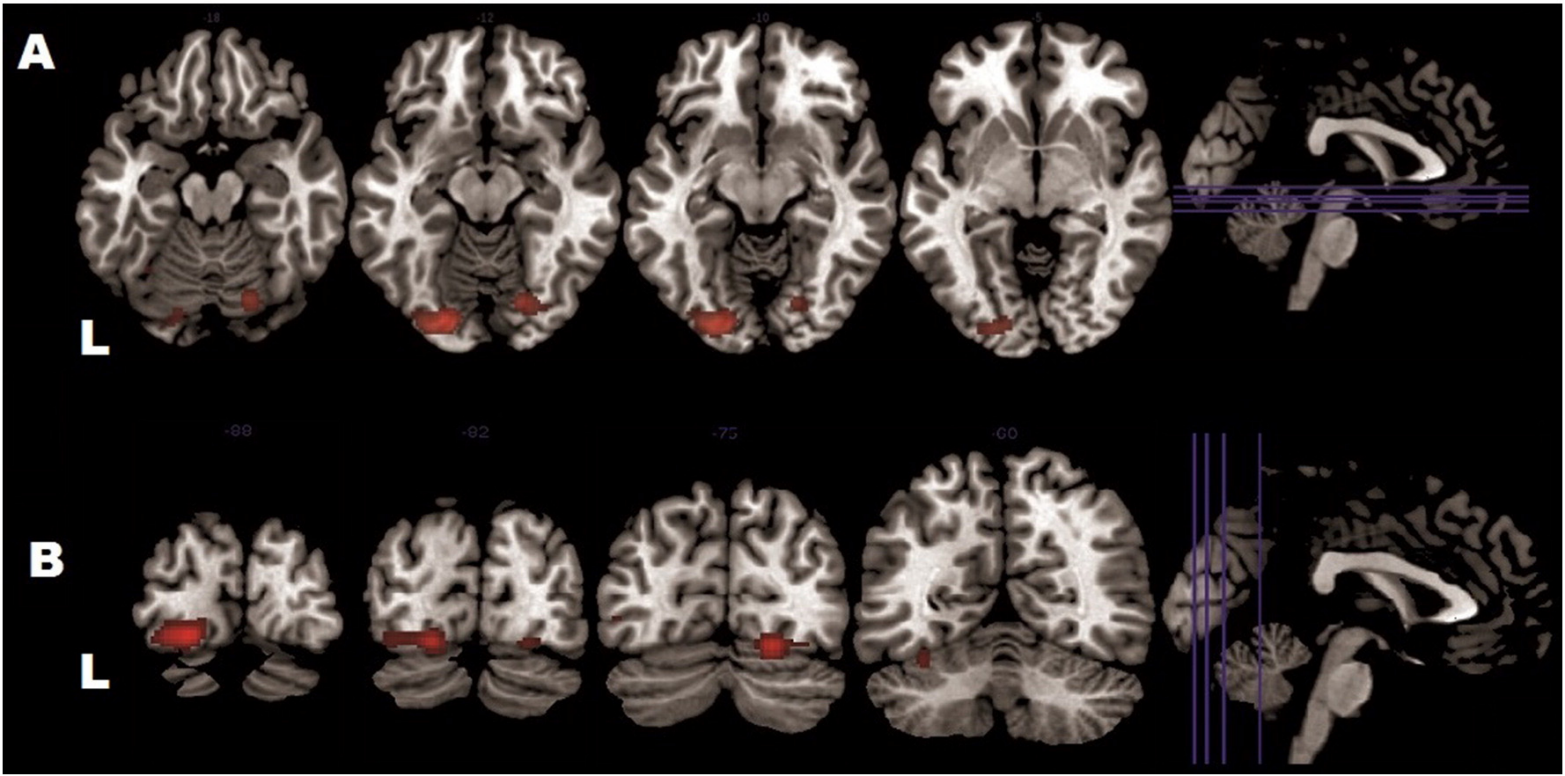
Exagereted visual response in BPD + MDD as compared to controls and MDD patients in bilateral lingual and the right fusiform gyri A. Axial view B. Coronar view The results are based on a conjunction analysis across two contrasts (all stimuli in BPD + MDD > all stimuli in controls ∩ all stimuli in BPD + MDD > all stimuli in MDD) each at p < 0.05 family-wise error (FWE)-corrected on the voxel level with corrected with a cluster extent of > 10 voxels.

**Fig. 6:**
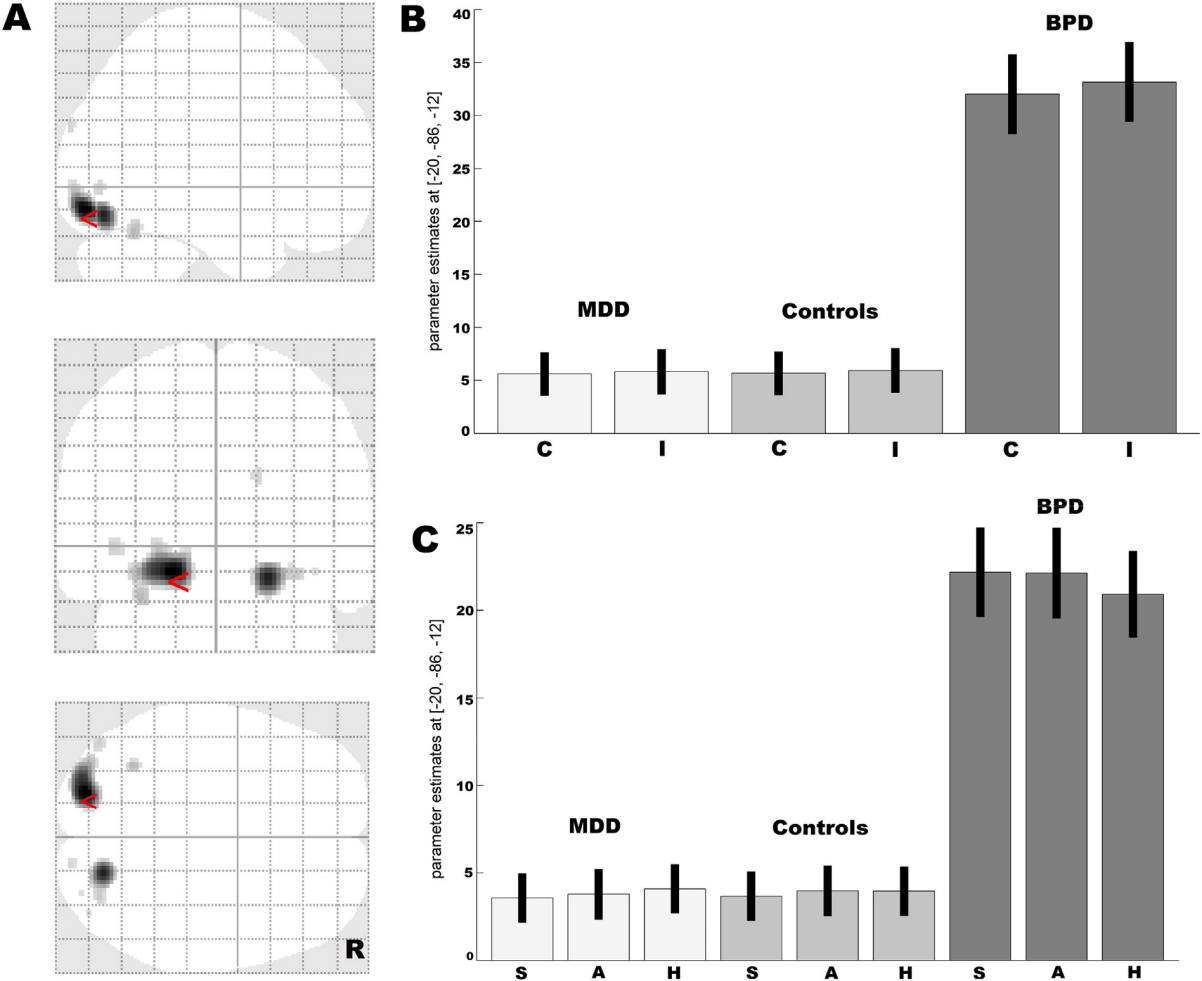
Visual response in BPD irrespective of congruency/emotion type of facial stimuli A. Glass brain showing exagereted visual response in BPD + MDD (as compared to controls and MDD patients) in the bilateral lingual and the right fusiform gyri B. Parameter estimates from the left lingual gyrus (peak MNI: − 20/− 86/− 12) showing that in the emotional Stroop task, BPD + MDD patients showed increased neural visual activity (as compared to healthy controls and MDD patients) irrespective of the congruency type or emotion type of stimuli. The results are based on a conjunction analysis across two contrasts (all stimuli in BPD + MDD > all stimuli in controls ∩ all stimuli in BPD + MDD > all stimuli in MDD) each at p < 0.05 family-wise error (FWE)-corrected on the voxel level with corrected with a cluster extent of > 10 voxels.

Collapsing across emotional face types irrespective of the congruency type, we noticed that participants with BPD + MDD uniquely displayed greater activation of the bilateral extrastriatal visual cortex, including the bilateral lingual gyrus and the right fusiform gyrus, compared to healthy controls and patients with MDD. The effect was seen in the conjunction analysis including two contrasts (all emotional stimuli in BPD + MDD > all emotional stimuli in HCs and all emotional stimuli in BPD + MDD > all emotional stimuli in MDD) (Table 2, Fig. 5). No other significant effects were observed in this contrast between the HC and MDD patients. In addition, no areas were more strongly activated in this contrast in HC or MDD patients compared to patients with BPD + MDD. Parameter estimates from the peak maxima in the left lingual gyrus (peak MNI: − 20/− 86/− 12; p < 0.001; *T* = 10.33; 406 voxels) demonstrate that the effects were not influenced either by interference or the emotional target type (Fig. 6).

#### 3.2.4 Correlation between behavioral interference and neuroimaging data

In healthy controls, the interference effect correlated with a network that included the pMFC and the bilateral superior and middle temporal cortices (Table 3), with no negative correlation being observed. Also, no significant correlation between the strength of the interference effect and neuroimaging data was observed in either patient group. The correlation analysis of the BOLD response to interference effect and psychopathology in the patient groups did not reveal any significant effects.

## 4 Discussion

We examined behavioral and neural responses to emotional conflict tasks in patients with borderline personality disorder with co-occurring major depression as well as patients with major depression with the aim to identify disorder-specific characteristics of BPD. In all three groups, emotional distractors generated a significant interference effect, defined as a slowing of RT from congruent to incongruent trials. Compared to the HCs, both patient groups performed more slowly without showing any differences in terms of behavioral interference effect.

At the neural level, in response to emotional targets independent of their emotion type (baseline contrast), the BPD + MDD group displayed, clearly and uniquely, a robust involvement of the visual areas (the right fusiform face area, the left middle occipital gyrus and the bilateral lingual gyri) and the cerebellum, regions that are central to the neural network responsible for early perception of emotional faces regardless of their emotional salience (Adolphs, 2002, Fusar-Poli et al., 2009). These regions (e.g. the fusiform face area) happen to be involved in face processing in general, irrespective of the emotional significance of a face. Thus, our observation may also indicate general deficits in the processing of facial stimuli in BPD as opposed to specific deficits in the processing of emotional faces.

Previous neuroimaging studies have also reported disturbances in cortical visual perception in BPD, with patients showing increased preparatory visual activity in the extrastriate cortex during anticipation of negative pictures (Scherpiet et al., 2014), heightened activity in the visual occipitotemporal cortical areas during an emotion discrimination task (Guitart-Masip et al., 2009) and greater amygdala and extrastriate cortex activity in the processing of negative social emotional pictures (Koenigsberg et al., 2009) along with an inability to acclimate, unlike healthy subjects, in the amygdala and fusiform gyrus when repeatedly exposed to interpersonal scenes (Koenigsberg et al., 2014). Our study shows disturbances in BPD in a large array of neural structures involved specifically in early perception of emotional faces. Remarkably, hyperresponsive visual activity toward emotional targets (faces) was not affected either by their congruency or emotion type. Thus, the effect was seen at the baseline level, appearing to be unrelated to the co-occurring depression, and representing specific dysfunctions/deviations in BPD, likely reflecting extreme sensitivity and reactivity to facial stimuli.

Among healthy controls, a parallel increase of conflict-related activity was seen in the VLPFC, the pMFC, and the parietal and extrastriate cortices, brain areas that are part of a network responsible for attentional control over emotional distractors, conflict detection and resolution (e.g. Chechko et al., 2012, Chechko et al., 2013, Dolcos and McCarthy, 2006, Egner and Hirsch, 2005, Harrison et al., 2005, Izuma et al., 2015). Higher levels of behavioral distractibility, defined as difference in RTs between incongruent and congruent trials in the HC, correlated with a stronger involvement of the pMFC, the bilateral superior temporal and the bilateral supramarginal gyri, brain regions related to cognitive emotion regulation (Kohn et al., 2014), suggesting that HCs (as opposed to both patient groups) adjusted the involvement of conflict processing recourses depending on conflict demands.

Compared to HCs, both patient groups displayed a reduction in conflict-related activity. In both patient groups, this effect was seen in the right VLPFC/insula region, owing to the fact that, unlike their healthy counterparts, the BPD + MDD and MDD patients did not involve the VLPFC in response to conflict trials (*incongruent* > *congruent trials* contrast).

In the MDD group, reduction of conflict-related activity was seen in the pMFC and the left inferior parietal lobule in addition to the right VLPFC/insula region.

The VLPFC/anterior insula region has been linked to action inhibition (e.g. inhibition of emotional distraction (Dolcos and McCarthy, 2006), attention reorientation (for review see Levy and Wagner, 2011) and detection and attribution of salience (Kohn et al., 2014). Clinical studies have underscored the involvement of the VLPFC in effortful affect regulation in patients suffering from bipolar affective disorder, with bipolar patients having been shown to not activate the VLPFC when regulating negative emotion to the same extent as healthy controls (Townsend et al., 2013). Initiated by the VLPFC, cognitive control over emotions is suggested to be executed by the pMFC (Kohn et al., 2014), a region formed by the posterior medial frontal gyrus, the supplementary motor area (SMA), the pre-SMA and the cingulate cortex (Amodio and Frith, 2006). Low neuronal activity in the VLPFC and the DLPFC has also been associated with poor cognitive regulation strategies in BPD (for review see Herpertz et al., 2014).

Collectively, our results indicate differences between HCs and both patient groups on multiple regulatory levels, starting from the evaluation of emotional salience to the initiation and execution of cognitive regulation. These abnormalities are likely linked to general irregularities in the processing and regulation of emotions, namely the vulnerability factor (Ehring et al., 2010) and core symptoms in BPD and MDD (Drevets et al., 2008, Krause-Utz et al., 2014). Deficits in the VLPFC are well documented also in bipolar affective disorder (Corbalán et al., 2015, Townsend and Altshuler, 2012), a condition similar to BPD in the clinical manifestation of mood lability and impulsivity (Ghaemi et al., 2014) and to MDD in terms of depressive episodes. Common irregularities in the cognitive control circuitries (especially in the VLPFC) in mania, depression and borderline personality disorder suggest that these findings are less disorder-specific, corroborating the assumption that BPD and MDD are emotion dysregulation disorders (Drevets et al., 2008, Krause-Utz et al., 2014). The ventrolateral prefrontal cortex dysregulation paralleled by disturbances in the cortical visual perception areas was, on the other hand, seen specifically in the BPD patient group. Clinically, BPD patients are known to be more susceptible to their own and other people's emotions, with difficulties in regulating them and placing them correctly. The resultant social dysfunction best distinguishes these patients clinically from other patient groups (Herpertz et al., 2014).

Finally, a couple of limitations of the study ought to be pointed out. First, the BPD group had marked clinical heterogeneity with a high comorbidity. Secondly, the healthy controls and BPD + MDD patients were significantly younger than their MDD counterparts. Besides depression, BPD typically coexists with anxiety, eating disorder and substance abuse, with only a small number of cases without comorbidity (Grant et al., 2008). The symptom severity of comorbid conditions can often obfuscate the diagnosis of personality disorder. In addition, the clinical manifestation of BPD, like all personality disorders, occurs early. Although MDD can occur at any age, the average age of developing MDD is 25 (Fleisher and Katz, 2001), hence the young age of our BPD group.

While the two patient groups showed some common differences in comparison to healthy controls with regard to the involvement of emotion-related neural circuitries, they did not differ in terms of behavioral performance, which is an additional limitation of the study. Furthermore, there were no significant differences between the patient groups with regard to behavioral performance and the involvement of emotion-related neural circuitries. Unfortunately, it is also not possible to determine whether the absence of a significant difference reflects the fact that the BPD group (or at least the BPD + MDD group) does not differ from the MDD in terms of the emotion processing mechanism, or whether the sample size (only 13 MDD + BPD subjects) was too small to yield an effect. Another limitation of the study is the lack of a pure BPD group, which, without MDD comorbidity, could help distinguish neural processes arising additively as a result of BPD co-occurring with MDD from those ensuing from the interaction of BPD and MDD processes in the same individual. Also, both patient groups differed in terms of medication, with the majority of the BPD + MDD group receiving a mood stabilizer in addition to antidepressants.

Despite the above limitations, this event-related fMRI study is the first of its kind to investigate the emotional Stroop interference effect between emotional face (target) and emotional word (distractor) in patients with BPD. Furthermore, to date, it is the first functional neuroimaging study to compare BPD patients with co-occurring depression with MDD patients, controlling for the co-occurring affective symptoms. Given that the overall depression levels were identical in both patient groups, the study also controlled for symptom severity, rendering its findings particularly robust with regard to disturbances in early perception of emotional faces in BPD. Comparing neuroimaging data obtained from BPD with those from other personality disorders, and axis I disorders such as bipolar spectrum or PTSD, can shed further light on the common and disorder-specific characteristics of these diseases.

## Funding source

The study was not funded.

## Conflict-of-interest declaration

The authors have no conflicts of interest to declare.

## Author contributions

Conceived and designed the experiments: NC, UH. Prepared ethical approval and recruited participants: NC, MA. Performed the experiments: NC, MA, MZ. Analyzed the data: NC, TK, MA. Wrote the paper: NC. Reviewed the manuscript: UH, TK, MZ, FS. Supervised the study: UH, FS.

## Figures and Tables

**Table 1:** Demographic and clinical characteristics of the groups.

	CONTROLS	BPD + MDD	MDD
Demographic data			
N (male)	24 (10)	13 (2)	21 (5)
Age, years	26.6 ± 3.2	24.6 ± 5.0	36.5 ± 10.8^a^
BDI, mean score	1.4 ± 0.8	31.0 ± 8.2	33.6 ± 8.1
HAMD, mean score	–	–	22.9 ± 4.8

ICD-10 classification of index episode, number of patients			
Major depression, single episode (ICD-10 F32)		13	21
Moderate depressive episode			3
Severe depressive episode			1
Recurrent major depression (ICD-10 F33)			
Mild depressive episode		3	1
Moderate depressive episode		6	7
Severe depressive episode		4	9
Age at depression onset, years		18.1 ± 3.3	29.7 ± 7.9^b^
Unmedicated patients, (percentage)		0	6 (23.8%)

Medication, MDD			
Free of medication			6
SNRI (venlafaxine or duloxetine)			8
SSRI (citalopram or sertraline)			5
Combination of bupropion, mirtazapine and trimipramine			1
Promethazine			1

Medication, BPD + MDD			
Free of medication		0	
SSRI (citalopram or fluoxetine)		4	
Combination of SSRI and valproate		2	
Combination of venlafaxine and aripiprazole		3	
Combination of tranylcypromine and lamotrigine		1	
Combination of bupropion and mirtazapine		1	
Combination of quetiapine and mirtazapine		1	
Combination of quetiapine, duloxetine and naltrexone		1	

Note.

There were no differences between the two patient groups in the severity depressive symptoms as assessed by BDI (p = 0.23; 31.0 ± 8.2 vs. 33.6 ± 8.1 in BPD's vs. MDD patients).

a Group of MDD was significantly older then healthy controls (p < 0.001; 36.5 ± 10.8 vs. 26.6 ± 3.2) and patients with BPD + MDD (p < 0.001; 36.5 ± 10.8 vs. 24.6 ± 5.0).

b Group of BPD + MDD were significantly younger at the moment of the onset of the first depressive episode (p < 0.001; 18.1 ± 3.3 vs. 29.7 ± 7.9 in BPD's vs. MDD patients).

**Table 2:** Increased visual response in BPD + MDD as compared to the HC's and the patients with MDD: conjunction analysis including two contrasts (all emotional stimuli in BPD + MDD > all emotional stimuli in controls and all emotional stimuli in BPD + MDD > all emotional stimuli in patients with MDD) each at the p < 0.05 FWE corrected with a cluster extend of > 10 voxels.

Anatomical region	Side	k	Peak voxel			
			T	X	Y	Z
Linual gyrus	L	406	10.33	− 20	− 86	− 12
Linual gyrus	R	191	9.27	20	− 76	− 14
Fusiform gyrus			5.27	32	− 78	− 14
Cerebelum	L	25	5.70	− 34	− 60	− 22
Middle occipital gyrus	L	15	5.18	− 46	− 78	− 2

**Table 3:** Positive correlation between BOLD response to emotional conflict and the strength of behavioral emotional interference effect in HC's at p < 0.05 FEW corrected on the cluster level with cluster-forming threshold of p < 0.001.

Anatomical region	Side	k	Peak voxel			
			T	X	Y	Z
Superior temporal gyrus	R	386	5.25	58	− 32	12
Superior temporal gyrus			4.68	48	− 34	8
Middle temporal gyrus			4.63	60	− 44	8
Posterior medial frontal gyrus	R	302	5.98	10	2	68
Posterior medial frontal gyrus			5.96	14	− 4	62
Superior frontal gyrus			4.43	20	4	60
Supramarginal gyrus	L	240	5.83	− 62	− 42	28
Superior temporal gyrus			4.65	− 52	− 40	
Middle temporal gyrus			4.42	− 58	− 44	20 10
Middle frontal gyrus	L	171	4.70	-30	12	54
Middle frontal gyrus	R	122	4.91	36	46	36
Superior frontal gyrus			4.18	28	44	20
